# Typho-Malarial Fever

**Published:** 1889-07

**Authors:** Jno. W. Trader

**Affiliations:** Sedalia, Mo.


### TYPHO-MALARIAL FEVER.

By JNO. W. TRADER, M. D., SEDALIA, MO.

Treatment.—Assuming that we have a case presenting the features of typho-malarial fever, we will adopt the plan of fortifying, as much as possible, all weak points and defending or attacking those that are most violently assailed by the oncoming malady.

As before stated, in our somewhat incomplete etiological description, we will find the peculiar tongue. The pulse is full and bounding, and, at the same time, rather soft with a gaseous feel, or what some call a dicrotous pulse—a sensation as though the artery rebounded at every beat.

The patient is dull—but little disposition to talk, and would rather be left alone. Notwithstanding we find the pulse one hundred and two or one hundred and three for the first few days.

Liver, stomach and spleen somewhat enlarged and tender, nausea and vomiting always present and at times very annoying and hard to control. A thin pale diarrhœa with flocculent particles floating about. Urine scanty and highly colored, at times, and again changing, as the disease advances, to light amber. Occasionally bilious matter is vomited, but not often.

We will now prescribe for the patient and see him again in twelve hours.

| | | |
|---|---|---|
| ℞ | Carbolic acid cryst | gr. viij. |
| | Squibb's tinct. opii | f ʒv. |
| | Glycerine | f ℥j. |
| | Aqua menth. pip., q. s. | ad. ʒiv. |

M. Sig. Teaspoonful every hour until the stomach settles, then give 10 gr. antifebrin every three hours until fever subsides.

We find upon our return that after the third dose of the above mixture the patient ceased to vomit, and has had only one action on the bowels. The nurse informs you that after the powder was given the patient went into a profuse sweat and seemed very much better in every particular. But you will find that the disease has simply been surprised, and, as a matter of course, had its forces somewhat demoralized, and the truce you now have is only a slight and momentary cessation of hostilities to enable the enemy to gather together and reform for another and more deadly attack. The pulse is one hundred; skin moist; temperature normal; patient quiet—as though feeling the effects of the opiate. Tongue still dry, red and pointed. We conclude that the patient is doing as well as could be expected, so we order the medicine continued, as the case demands, only giving the drops if he becomes restless, and another powder should the fever arise. We will see him again in the morning. But now comes the very unpleasant experience of every one, I suppose, who ever practiced medicine. About one o'clock a. m. the telephone begins to ring as though it would "tear the house down," and when you, shivering in deshabille, put the phone to your ear and halloo, you are requested to come out to Mr. B——— just as soon as you can.

You find your patient raving—almost impossible to keep him in bed—the friends very much alarmed, and, as a consequence, of but very little aid to you in this battle. The bowels have moved unconsciously, the nose has been bleeding, and, altogether, you have a unpleasant state of affairs. The patient recognizes you and yields to your authority. You order the soiled linen removed while you prepare the following:

| | | |
|---|---|---|
| ℞ | Brom. potass. | gr. xxx. |
| | Squibb's Ergot | f ʒss. |
| | Squbb's tinct. opii | gr. xv. |
| | Aqua font. | ℥ ss. |

M. Sig. To be given at once.

You now take your seat by the bedside and request all the members of the family, except the father, to retire. The patient tries to get up, but your firmness and authority soon quiets him, and in twenty minutes he is asleep and resting easy. Let him rest. In about an hour you give him half teacupful of beef tea, and, if necessary, a teaspoonful of good brandy or whisky, and let this plan of nourishment be kept up at regular intervals of one or two hours, and give the carbolic acid mixture every hour or two, or often enough to quiet all nervous disturbance and control diarrhœa. Give a powder of antipyrin every six hours, beginning at two o'clock p. m. and ceasing at two a. m.

Now that all is still, you can have time for a little reflection. Suppose that I had followed up the medicine with more promptness, after the first abatement of the symptoms, would it not have been better? Most assuredly. Here is where the mistake is too often made.

You must not expect to break up, or cut short, an attack of fever of this kind. You must *conduct* it to a favorable termination and not try to *cure* it too soon.

Do not give any quinine until the tongue becomes moist and broadens out, when extended, and the point lies well down towards the chin.

Localized organic trouble must be watched for and remedied at the onset. Do not become too dogmatic in your treatment and *never* use the hypodermic syringe in these cases. Remove all sources of irritation, as far as possible, and, while you must be firm and positive in what you do and say, let your conduct, at all times, be tempered with that best of all graces, a genuine Christian charity.—*St. Louis Medical Journal.*

